# Resilience Testing of Health Systems: How Can It Be Done?

**DOI:** 10.3390/ijerph18094742

**Published:** 2021-04-29

**Authors:** Heather L. Rogers, Pedro Pita Barros, Jan De Maeseneer, Lasse Lehtonen, Christos Lionis, Martin McKee, Luigi Siciliani, Dorothea Stahl, Jelka Zaletel, Dionne Kringos

**Affiliations:** 1 Biocruces Bizkaia Health Research Institute, 48903 Barakaldo, Spain; 2 IKERBASQUE Basque Foundation for Science, 48009 Bilbao, Spain; 3 Nova School of Business and Economics, Universidade Nova de Lisboa, 2775-405 Lisbon, Portugal; ppbarros@novasbe.pt; 4 Department of Public Health and Primary Care, Ghent University, 9000 Ghent, Belgium; jan.demaeseneer@ugent.be; 5 Helsinki University Hospital and University of Helsinki, 00029 Helsinki, Finland; lasse.lehtonen@hus.fi; 6 Clinic of Social and Family Medicine, School of Medicine, University of Crete, 71003 Heraklion, Greece; lionis@galinos.med.uoc.gr; 7 Department of Health Services and Policy, London School of Hygiene & Tropical Medicine, London WC1H 9SH, UK; martin.mckee@lshtm.ac.uk; 8 Department of Economics and Related Studies, University of York, York YO10 5DD, UK; luigi.siciliani@york.ac.uk; 9 Klinikum Bielefeld, Universitätsklinikum OWL der Universität Bielefeld, 33604 Bielefeld, Germany; dorothea.stahl@klinikumbielefeld.de; 10 National Insitute of Public Health Slovenia and University Medical Center, 1000 Ljubljana, Slovenia; jelka.zaletel@kclj.si; 11 Amsterdam UMC, Department of Public and Occupational Health, Amsterdam Public Health Research Institute, University of Amsterdam, 1105 AZ Amsterdam, The Netherlands; d.s.kringos@amsterdamumc.nl

**Keywords:** COVID-19, health system, resilience testing

## Abstract

The resilience of health systems has received considerable attention as of late, yet little is known about what a resilience test might look like. We develop a resilience test concept and methodology. We describe key components of a toolkit and a 5-phased approach to implementation of resilience testing that can be adapted to individual health systems. We develop a methodology for a test that is balanced in terms of standardization and system-specific characteristics/needs. We specify how to work with diverse stakeholders from the health ecosystem via participatory processes to assess and identify recommendations for health system strengthening. The proposed resilience test toolkit consists of “what if” adverse scenarios, a menu of health system performance elements and indicators based on an input-output-outcomes framework, a discussion guide for each adverse scenario, and a traffic light scorecard template. The five phases of implementation include Phase 0, a preparatory phase to adapt the toolkit materials; Phase 1: facilitated discussion groups with stakeholders regarding the adverse scenarios; Phase 2: supplemental data collection of relevant quantitative indicators; Phase 3: summarization of results; Phase 4: action planning and health system transformation. The toolkit and 5-phased approach can support countries to test resilience of health systems, and provides a concrete roadmap to its implementation.

## 1. Introduction

The COVID-19 pandemic has put national health systems under immense pressure. Health systems throughout the world demonstrated different levels of preparedness for an outbreak of this magnitude. The crisis tested the ability and capacity of health systems to absorb, effectively respond and adapt to shocks and structural changes while sustaining day-to-day operations; in other words, it tested ‘health systems’ resilience’. There are various definitions of resilience. A frequently cited definition, offered by Kruk and colleagues (2015), defines health system resilience as “the capacity of health actors, institutions, and populations to prepare for and effectively respond to crises; maintain core functions when a crisis hits; and, informed by lessons learnt during the crisis, reorganize if conditions require it” [1].

Health system resilience has been on policy agendas for many years [2]. Most recently, prior to COVID-19, the 2019 State of Health in the EU country reports highlighted longstanding concerns about resilience. Yet, it was only after the COVID-19 outbreak that country-specific recommendations on health systems resilience have been made to each Member State in the European Semester process [3]. Numerous analyses have already been published on the impact of immediate crisis response measures (e.g., [4,5,6]). Notwithstanding this extensive literature, there is a need to look beyond the current pandemic and identify new frameworks and policy tools that affect health care organization in order to better prepare for future crises and other challenges that may affect health care delivery.

Testing the resilience of health systems has also received considerable attention as of late as a potential approach to strengthen health care systems [7]. However, there is limited literature to guide research development of the application of a resilience test or what it might look like in practice. Yet, since the 1990s, resilience testing has been used in the banking sector, in the form of internal stress tests. The health care sector can build on such experiences. In a ‘resilience test’ of health systems, shocks or structural changes could be introduced in ‘what if’ scenarios [8]. Hypothetical responses to the shock could be examined to identify strengths and weaknesses in system performance under potential stress. Following lessons learned from the example of bank stress tests, such resilience tests should value contextualized exercises over standardized ones, learn from the process of producing the results rather than the results themselves, and develop solutions to address failure [8]. The International Health Regulations of the World Health Organization offers a Joint External Evaluation Tool (JEE Tool, see: https://www.who.int/ihr/procedures/joint-external-evaluations/en/) to assess country preparedness and response capacities to public health risks. Limitations of the self-evaluation phase is that it occurs at the level of the ministries, which means the results provided for the external evaluation phase tend to be restricted to official sources of information. Both the concept and methodology for health system resilience testing need to be further explored in order to be useful for practical implementation leading to meaningful results.

In 2020, the European Commission requested its Expert Panel on Effective Ways of Investing in Health (Expert Panel), comprising 17 experts from across the EU, to operationalize the concept of resilience testing of a health system [9]. Building on that work [10], this paper further develops the resilience test concept and methodology, enhancing the description of key components of a toolkit that could be adapted to individual health systems and a 5-phased approach to implementation of resilience testing. First, we provide a definition of resilience and propose an appropriate framework for the examination of resilience in health systems. Second, we illustrate the use of the framework in the context of a resilience test. Third, we detail the main components of the resilience test toolkit, which can be adapted to different health systems. Lastly, we propose a roadmap for resilience test implementation. The discussion section reflects upon these advancements and offers directions for future research.

We contribute to the previous literature by refining the definition of resilience, which maps into a multidimensional framework for health and social care, and developing a pragmatic set of tools and guidelines for resilience test structure and implementation. We go beyond existing literature by specifying methods to work with diverse stakeholders from the health ecosystem via participatory processes to assess and identify recommendations for health system strengthening.

## 2. Materials and Methods

The present paper builds and extends work conducted by the Expert Panel [9]. A literature review on the concept of resilience in health systems and experiences with resilience testing in other sectors was conducted (e.g., banking). A subgroup of the Expert Panel (authors) explored concept definitions, frameworks, methodologies, early experiences and lessons learnt on health system resilience during the first wave of the COVID-19 pandemic, drawing on the scientific and grey literature including reports from international organizations (e.g., OECD, WHO), networks and working groups (e.g., EU Expert Group on Health Systems Performance Assessment). H.L.R., P.P.B., J.D.M., L.L., D.S., and J.Z. held four monthly meetings between June and September 2020 to discuss and critically appraise the literature and draft and review text. H.R. drafted text based on discussion for review and consensus. This work was shared with the larger drafting group (see Acknowledgements section) and the responsible technical officers of the European Commission’s Directorate General on Health and Food Safety (DG SANTE). Draft versions of the opinion were shared with the broader Expert Panel in plenary meetings to incorporate their professional experiences in health system policy, practice and research. The resulting opinion was discussed in a public hearing and improved accordingly.

## 3. Results

### 3.1. Definition of Resilience and an Underlying Framework for Its Assessment in Health Systems

#### 3.1.1. Definition

A practical definition of resilience is the first requirement at the core of the resilience test. Resilience of health system addresses three main capacities: absorptive, adaptive, and transformative [11]. The absorptive capacity relates to the capacity of a health system to continue to deliver the same level (access, quality and equity) of health care services and protection to populations despite the shock using the same level of resources and capacities. Adaptive capacity is the capacity of the health system actors to deliver the same level of health care services with fewer and/or different resources, which requires making organizational adaptations. The transformative capacity describes the ability of health system actors to transform the functions and structure of the health system to respond to a changing environment.

Using these elements, the Expert Group on Health Systems Performance Assessment provide a useful working definition of resilience, emphasizing the importance of the transformational capacity of the health system [12]: “Health system resilience describes the capacity of a health system to

(a) proactively foresee;
(b) absorb; and
(c) adapt to shocks and structural changes in a way that allows it to
  (i) sustain required operations;
  (ii) resume optimal performance as quickly as possible;
  (iii) transform its structure and functions to strengthen the system; and (possibly)
  (iv) reduce its vulnerability to similar shocks and structural changes in the future”.

The implication of this definition for resilience testing is the need for system transformation to ensure optimal health system functioning in the long term.

#### 3.1.2. Framework

**Figure 1:**
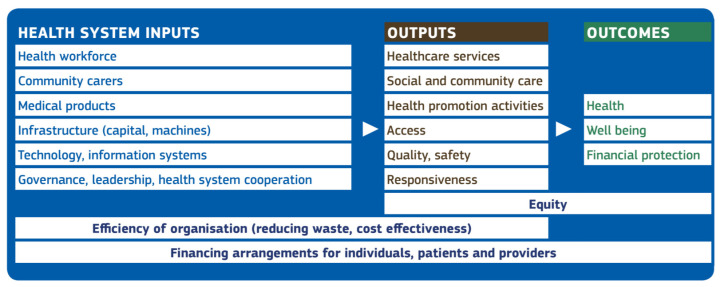
Multi-dimensional Health and Social Care Systems (MHSCS) conceptual framework. Source: Expert Panel Opinion [10].

A common conceptual framework of resilient health systems is required to develop a resilience test. In the Opinion [10], The Expert Panel developed the Multi-dimensional Health and Social Care Systems (MHSCS) conceptual framework that builds on previous ones [13,14,15]. In 2009, the World Bank, the Global Alliance on Vaccines Initiative (GAVI) and the Global Fund to Fight AIDS, Tuberculosis and Malaria (GFATM) offered a monitoring and evaluation framework to assess strengthening of health systems and a focus on data collection. This framework uses the inputs-outputs-outcomes approach [13]. In 2010, the World Health Organization (WHO) identified six ‘building blocks’ of a health system: health service delivery, health workforce, health information systems, access to essential medicines, health systems financing, and leadership and governance [14]. Lastly, in 2018, Sacks and colleagues extended the WHO 2010 ‘building blocks’ model to include community services [15], as community organizations and societal partnerships make important contributions to health outcomes. The MHSCS conceptual framework presented in Figure 1 combines and extends the elements from these three frameworks. It uses an inputs-outputs-outcomes structure to illustrate the relationships among key elements that contribute to viable and resilient health systems that support the Sustainable Development Goals [10]. See Figure 1.

Elements of the MHSCS conceptual framework feature within and across the inputs-outputs-outcomes structure. Health workers and community carers are health system critical “inputs” that must be supported by adequate infrastructure (buildings, primary and secondary care facilities, equipment) and information systems. Governance and leadership help to ensure that everyone works towards a common goal, including cooperation across health systems. Among the “outputs” of the health system, health and community workers deliver health services, social and community care, and health promotion activities. Furthermore, health services must be accessible, of high quality, and responsive to patient needs. In terms of “outcomes”, health services contribute to the health and well-being of patients and individuals, and the rules governing the access to such services (e.g., the absence of co-payments) determine financial protection. Equity is a ubiquitous health system objective, but inequities frequently persist both in the health care delivery (“output”) and in health or other “outcomes”. Efficiency, through improved organization and delivery of health and community services (“outputs”), can also improve “outcomes” through better access, quality and responsiveness (“outputs”). Financing arrangements affect both users and providers of health care. Existing and potential future patients contribute to financial protection by financing the health system through taxes and social insurance contributions. In turn, the resources collected are redistributed to providers under a wide range of financial arrangements.

### 3.2. Application of the Multi-Dimensional Framework in a Resilience Test of Health Systems

**Figure 2:**
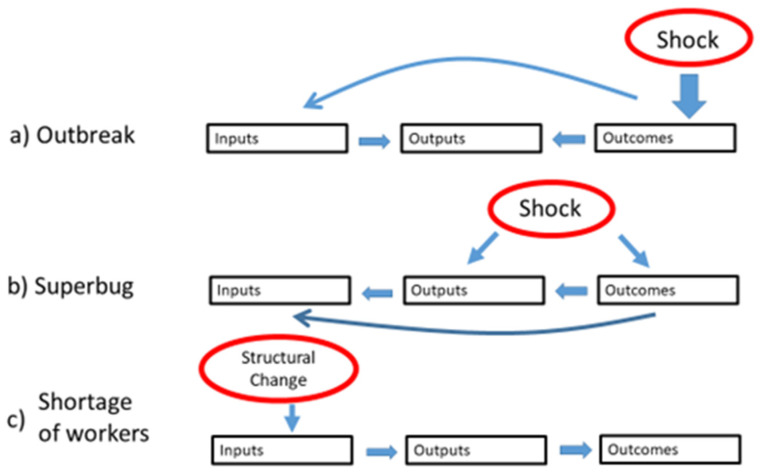
Hypothetical responses of a health system to example shocks or structural changes labelled (**a**–**c**). Source: Expert Panel Opinion [10].

In a resilience test, shocks or other major structural changes are introduced into the health system using ‘what if’ scenarios. Then, hypothetical responses to the shock are examined by stakeholders to identify strengths and weaknesses in system performance under plausible stressors. Similar to a prudential stress test of banks, a resilience test on a health system implies that the interest is in system-wide effects, not on the impact on specific health care institutions and how they individually cope in adverse scenarios. In other words, it tests the health system as a whole, with its inter-connected parts, which is often more than the sum of the impacts on individual entities. The inputs-outputs-outcomes structure of the MHSCS conceptual framework is considered to be appropriate for this type of assessment and can be used to examine immediate and potential downstream effects of a shock to the system. As described in the Opinion [10], the MHSCS conceptual framework provides a common map that can be used to discuss mechanisms of action of hypothetical shocks in the context of a ‘resilience test’ of health and social care systems. See Figure 2.

Figure 2 illustrates how two different types of shocks and a structural change might hypothetically impact health care system inputs-outputs-outcomes. In example (a), an outbreak of an infectious disease affects population health (an “outcome” element), and the health system needs to respond through a change in the organization of the workforce and its resources (“input” elements), which will affect the delivery of the services (“output” elements), but will also impact directly on the ability to maintain the delivery of services (“outputs”). In example (b), a superbug caused by weak safety procedures in the delivery of hospital services (“output”) has an immediate effect on patient health (“outcome”), which in turn triggers corrective and containment measures in service delivery (“output”) and organization of the medical workforce (“input”). The chronic shortage of certain type of workers (“input”) can affect health system ability to deliver services (“output”) and improve health (“outcome”).

The MHSCS conceptual framework has both theoretical and practical relevance to the resilience test. First, it helps to crystalize thinking about the myriad ways in which a potential shock might impact system functioning. Second, the elements define broad categories of potential indicators to monitor and evaluate system functioning, both under stress and in absence of a shock. Lastly, the MHSCS conceptual framework can assist the stakeholders involved in the resilience test process in building strategic resilience. As the potential relationships between the elements are explored by stakeholders in the context of hypothetical shocks in ‘what if’ scenarios, system-specific recommendations for transformation can be developed, implemented, and assessed in order to ensure optimal health and social care system functioning in the long term.

### 3.3. Resilience Test Structure and Implementation Methodology

In the Opinion [10], a 5-phased resilience test was proposed with standardized toolkit components. The resilience test ends with an action planning and transformation phase in which health system capacities are strengthened to enhance resilience to future adverse scenarios. Each phase is briefly described below, with any special considerations noted. Throughout all of the phases of the resilience test process, the ability of the resilience test to generate relevant data is assessed. Continuous monitoring and evaluation allows for process improvement during the process, instead of after it has concluded. The toolkit was designed to ensure meaningful assessment, yet to be sufficient flexible that can be implemented across many different health systems with diverse institutional features.

**Figure 3:**
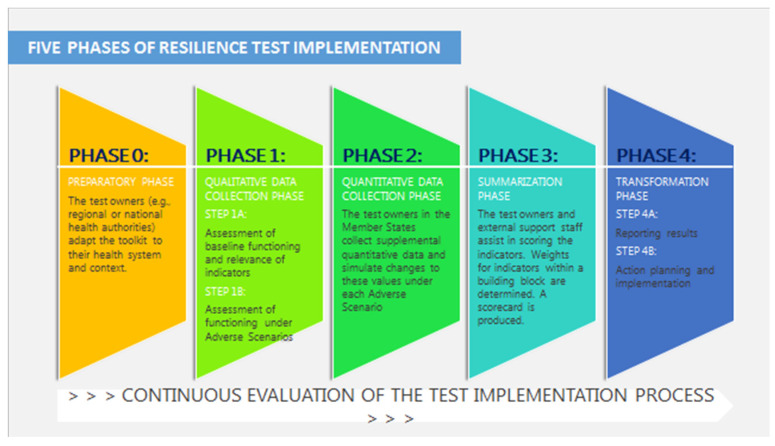
Five phases of resilience test implementation. Source: Modified from the Expert Panel Opinion [10].

A visual representation of the resilience test process and key actors is provided in Figure 3.

#### 3.3.1. Phase 0: Preparatory Phase

In this phase, the test owners, who are the health authorities responsible for the health systems (at the national or regional level), adapt the toolkit materials to their health system and context. Quantitative data to support the realistic development of the adverse scenarios is gathered. MHSCS elements and associated indicators hypothesized to be impacted by different adverse scenarios are defined. The discussion guides for interaction with health systems stakeholders are finalized. A trained facilitator must be designated to lead the discussion groups. This person may be from an external team supporting resilience testing across health systems, or may be a person from the regional/national health system who is trained to effective carry out the facilitator functions.

#### 3.3.2. Phase 1: Qualitative Data Collection

Step 1A—Assessment of baseline functioning and relevance of indicators: Stakeholders convene in groups of eight based on their role in the health system (e.g., type of health care provider, manager, user, and inter-sectoral collaborator). The facilitator uses the adapted toolkit to lead stakeholders through a discussion examining the normal and natural evolution of health system functioning in the absence of any particular stressor (under “normal” conditions). The stakeholders address the extent to which each indicator is aligned with health system values and context. The information from this discussion is used for the stoplight scoring system in Phase 3.

Step 1B—Assessment of functioning under adverse scenarios: The facilitator presents each group of stakeholders the “what if” adverse scenario and elicits responses from the group as to the impact on the health system based on how the group members themselves would react or respond. The discussion guide from the toolkit that is specific to the adverse scenario is used. Tabletop exercises methodologies (see [16,17,18]), which include participatory leadership, Participatory Learning and Action, Design Thinking, and LEGO© Serious Play, facilitate in-depth analysis by the participants and lead to knowledge generation. Each group discusses the changes in the elements and relevant indicators that the health system would experience relative to baseline capacities. Both short-term and longer-term impact and responses are considered. For instance, as the scenario evolves over time, stakeholders examine the system response to actions taken, known as second-round effects.

Some general questions, relevant to any adverse scenario, include

1. What is the impact of the adverse scenario? Where does it impact in the health system?
2. What tools and resources are available to be exploited (e.g., databases, protocols, human resources)?
3. How will the adverse scenario be managed from an organizational perspective (e.g., organizational models, capacities of staff, organizational change)?
4. What aspects of the ecosystem (e.g., mental health, psycho-social impact, equity, human rights, social cohesion) will be monitored and how?
5. How will decisions be made and implemented?
6. How will different levels of care communicate and integrate?

When the discussion concerning the first adverse scenario has concluded, a second adverse scenario is presented to the group and a new discussion ensues. At least two adverse scenarios should be presented and assessed separately in this step to show varying responses.

#### 3.3.3. Phase 2: Quantitative Data Collection

Based on the Phase 1 discussions with stakeholders, health authorities identify and obtain available supplemental quantitative data on the indicators under “normal” conditions and are asked to simulate changes to these values in response to each adverse scenario. Additional discussion groups can be conducted as needed to ensure a comprehensive assessment of health system response under both adverse scenarios.

#### 3.3.4. Phase 3: Summarizing

The facilitator and members of the external support team synthesize the qualitative Phase 1 and quantitative Phase 2 information collected. They may be assisted by regional/national health authorities, and possibly by representatives from other health systems who have already completed the resilience test process. The synthesis involves quantifying the information gathered from the prior phases to produce a health system-specific scorecard. This entails assessing each of the relevant indicators identified for each relevant MHSCS element according to the Phase 0 and Step 1A results. Each indicator is rated on a 4-point Likert scale. Appropriate weights for each indicator of a given MHSCS element are determined by interpreting the data in Step 1A. The scorecard is generated.

#### 3.3.5. Phase 4: Reporting and Action Planning for Transformative Change

Step 4A—Reporting: Results are shared with stakeholders—both those who participated in the prior phases and other stakeholders who did not directly contribute information. Small groups of stakeholders convene and, led by the facilitator, engage in critical reflection on the results. These groups identify specific key areas where improvements are needed, and offer recommendations in the form of summative as well as formative evaluation. The results across groups are synthesized by the facilitator, external support staff, and possibly national health authorities and representatives from other health systems.

Step 4B—Action planning and implementation: This step is crucial for the development of strategic resilience, in other words, to achieve long-term changes to counteract the effects of similar adverse scenarios in the future. The regional/national health authorities identify, based on the results, an owner of the process of action planning and implementation. He/she should be a stakeholder with high interest and endurance, able to overcome obstacles, motivate others, and sufficient drive to follow-through on goals. This individual should have a certain level of power and a reasonable level of capacity to be able to transform the structures and functions in the health care ecosystem. The collaborative process that this individual will lead requires participatory leadership methods and expertise in consensus building. He/she will have to balance the potential impacts of various changes, as well as competing timeframes, feasibility, interests and power of the stakeholders involved, all while maintaining trust.

Based on the scorecard from Phase 3 and the recommendations from Step 4A, a collaborative process is led by the owner of this phase so that stakeholders can act on the MHSCS elements. Relevant facilitators and barriers to implementation of key recommendations are identified and strategies to strengthen or overcome them are developed. Qualitative assessments underlying the scorecard summary are reviewed for potential additional solutions.

In summary, the resilience test implementation process is a dynamic, iterative, and collaborative process in which test owners (e.g., national health authorities) engage diverse stakeholders from the health ecosystem. Stakeholders include individuals who design health systems and/or have strategic decision making capacity in the health system, which will help to ensure that the resilience test results lead to action planning for improvement. Stakeholders also include inter-sectoral collaborators that influence population health, e.g., by professionals in education and housing sectors. Emphasis in the implementation methodology is placed on inclusive, participatory strategies based on “proportionate universalism” that includes everyone but with a progressively greater emphasis on vulnerable or at-risk groups [19]. The collaborative process requires that stakeholders involved in the resilience test process have a safe environment in which to express their opinions without fear of reprisal. Test owners must be willing to listen to these views and offer stakeholders a voice. Similarly, test owners, in collaboration with external support staff, should foster trust among stakeholders. In essence, the test owners must demonstrate openness to feedback, including potential criticism, and a desire to improve. The test owners must have sufficient political, scientific and operational capacity to carry out the test in this manner.

### 3.4. Core Components of the Resilience Test Toolkit

The resilience test “toolkit” is a set of four standardized components that form the basis of resilience testing of health care systems. See Table 1. Each component has a process that allows it to be customized to each unique health system’s context, as described below. The Opinion [10] identified some of the these components. In this paper, we offer additional components and further elaborate and clarify the customization process.

#### 3.4.1. Component 1: Different Adverse ‘What If’ Scenarios

In order for the resilience test of health systems to be as accurate as possible, realistic adverse “what if” scenarios must be fully developed. Those scenarios which contain threats that health system stakeholders believe that they will have to face in the future will be the most effective resilience test scenarios. Because two scenarios are part of the resilience test implementation, the toolkit should have at least five different scenarios for the test owners to choose from. Table 2 describes the basic elements of one possible adverse scenario—that of a “super-bug” outbreak.

The toolkit includes instructions for the test owner in the health system to customize each adverse scenario to his/her context. Together with the external staff supporting the resilience test implementation process, the test owner adapts the scenario to make it more relevant for that particular health system. The “super-bug” scenario might be tailored to a particular health system by adding tables of data indicating how different hospitals in the country are being affected. A fictitious letter or report from the administration at an important hospital in the country and addressed to health authorities could specify their experience and request closure. Regarding the cleaning procedures, the scenario could include descriptions of three possible technologies that might be used, along with costs and timeline for procurement. A report describing the human resource capacities required for the clean-up could be added. Adding these details to make the ‘what if’ scenario realistic is likely to enhance the validity of the results for different health systems.

To further tailor the ‘what if’ scenario to different groups of stakeholders, the toolkit provides some initial areas of concern common across health systems with respect to the given scenario. These concerns tend to differ by type of stakeholder, and stakeholders within and outside of the health system should be considered. Each scenario is accompanied by a list of concrete questions so that country-specific characteristics can be fully incorporated as relevant. Some possible issues relevant to the “super-bug” scenario are highlighted in Table 2.

Initial discussions about the scenarios between the test owner and external support staff can allow for new elements to be scripted into the scenario as required by the particular health system. For instance, as a result of discussion the communication issues facing decision makers, the “super-bug” scenario text for this group might include supplementary text suggesting “Public opinion and polls ahead of a general election in 9 months has increased pressure on the Minister of Health to act decisively. In the cabinet of the Minister, a decision has to be made regarding centralizing decisions made concerning hospitals, as well as the communication strategy, or taking a decentralized approach, leaving decisions to individual hospitals. A task force has been created to advise on this issue.” Further discussions between the test owner and test collaborators might consider prior health system experience with other shocks that have actually occurred in the past. Together, the test owner and the external support staff might hypothesize mechanisms of action of the shocks presented in each of the five scenarios using the MHSCS conceptual framework elements in order to appropriately select the two scenarios for use in the resilience test implementation sessions. The purpose having multiple scenarios to choose from and being able to customize them is to specify the “right questions” that guide the test owner to examine the necessary aspects of system functioning under stress.

#### 3.4.2. Component 2: Menu of Key Indicators

The second core component of the resilience test toolkit is the menu of key indicators that correspond to the elements of the MHSCS conceptual framework. Numerous publications detail potential quantitative indicators of health system performance assessment, monitoring, and/or strengthening. A non-exhaustive list includes those published by the World Health Organization, the Organization for Economic Cooperation and Development (e.g., the EC-OECD report [20] examining how resilient European health systems have been to the COVID-19 crisis), reports by Eurostat and the European Observatory on Health Systems and Policies [21], as well as compilations in prior opinions by the Expert Panel [22]. Table 3 provides a potential selection of indicators that align with the elements in the MHSCS and are relevant for health system functioning. Indicators of function under “normal conditions”, e.g., in the absence of a particular shock, as well as a result of the shock to the system are defined. This distinction is useful because a health system may be able to effectively use existing knowledge and resources, but may struggle to use, adapt, or develop new knowledge and resources when the system is under shock.

It is important to note that indicators for a given element can be specific, objective and quantitative (such as the number of patients per medical professional) or more subjective and qualitative (for instance, the extent to which different specialties and disciplines are integrated within the health system). Both types of indicators provide important information with respect to health system functioning.

One possible approach to selecting relevant indicators can leverage realist evaluation, which is being used increasingly in health services research. Realist evaluation examines ‘What works for whom, in what circumstances and why?’ [23]. Discussions between test owners and external collaborators in the resilience test preparation phase can apply this realist approach to hypothesize context-mechanism-outcome (CMO) configurations under adverse scenarios [24,25]. The hypotheses that are generated can be used to extend the lists of health system-specific indicators that reflect actual functioning. The hypotheses help to ensure the selection of meaningful indicators of health system functioning, both in “normal” conditions and when a shock is introduced into the system.

#### 3.4.3. Component 3: Discussion Guide for Facilitation of Resilience Test Implementation Sessions

The third core component of the resilience test toolkit is the discussion guide that will used by the facilitator of the discussion sessions with stakeholders that are central to resilience test implementation. A discussion guide script outline for each MHSCS element and related to the menu of indicators will help stakeholders to consider system functioning both under “normal” conditions (in the absence of the shock) and after the shock hits the system. The discussion guide can consider the possible existence of a one-to-one relationship between indicators of functioning from “normal” to “shock”, as well as the comprehensiveness of the menu of indicators for each MHSCS element. Through discussion, additional indicators can emerge.

To illustrate how the discussion guide might be fully developed within the resilience test toolkit, the “super-bug” ‘what if’ scenario from Table 2 is expanded. As prerequisites, a fully elaborated, system-specific scenario has been established (core toolkit component 1) and key health system elements and indicators affected have been determined, potentially using CMO configurations (core toolkit component 2). As seen in Figure 2 example (b), the super-bug is purported to have an immediate impact on population health ‘outcomes’ and health care services ‘outputs’. Multiple MHSCS elements (‘inputs’) are affected downstream by the shock. The elements affected may vary across health systems, but can be hypothesized to include health workforce, information systems, and infrastructure. Governance also becomes especially critical in times of stress. Therefore, it would be essential to include these elements and related indicators in the discussion guide.

Discussion questions may differ depending on the stakeholder group being addressed (see Table 3 for examples of how the health system-customized scenario can be tailored). Table 4 suggests that example potential MHSCS indicators concerning the Health Workforce element means that discussion questions would be developed to center on the extent to which the health system adequately:

- Trains qualified professionals,
- Integrates different specialties and disciplines,
- Addresses mental health of professionals,
- Re-assigns health professionals,
- Engages in task shifting, and
- Expands responsibilities of health professionals.

In addition, available quantitative data at baseline would be collected on:

- Different types of professionals per population,
- Patients per type of health professional, and
- Integration schemes covering different types of patients and health professionals.

The participants would then be guided by additional questions to help them anticipate how these capacities and numbers might be impacted by the shock in the ‘what if’ scenario.

Similarly, questions on how the health services element may be affected by the “super-bug” scenario might include the extent to which the health system maintains access in line with health needs, including mental health care, and ensures access to care for vulnerable groups, as well as supports primary care services. In addition, the participants can discuss how certain quantitative indicators, such as waiting times for services, satisfaction ratings, and percent of the population without coverage might change over time as the system responds to the shock and adapts.

Each of the other relevant elements and key indicators (e.g., information systems, infrastructure, and governance) would be discussed in a similar manner by referring to the indicators and corresponding discussion questions.

#### 3.4.4. Component 4: Scorecard Template to Synthesize Results

**Figure 4:**
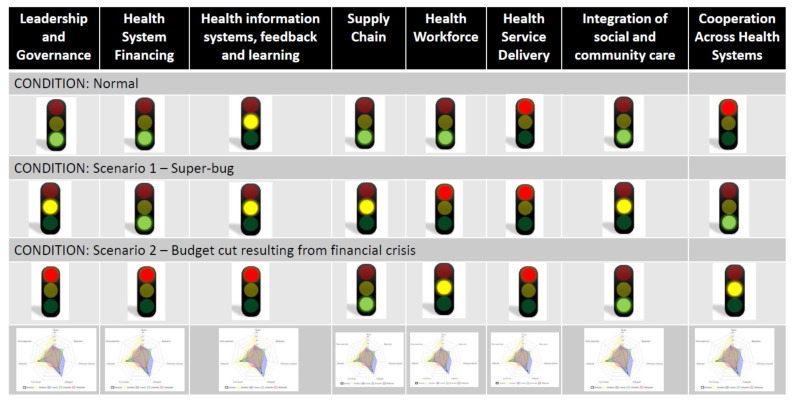
A sample scorecard to illustrate an overview of resilience test findings. The radar plots in the figure are for illustrative purposes only. Source: Expert Panel Opinion [10].

As means of feedback to the health system stakeholders and to facilitate further discussion on planned improvements, it is valuable to have a template to synthesize the information collected during the resilience test and offer an overview of health system functioning. One way to accomplish this is via a scorecard or dashboard with traffic lights for each MHSCS element based on the compilation of relevant indicators. The purpose of the scorecard is to offer a snapshot view of elements of health system functioning both under “normal” conditions and in each ‘what if’ scenario discussed as part of the resilience test implementation. A green light indicates that the element is functioning well in the given condition and is likely to weather the shock. Yellow suggests some deficiencies in that element and that caution is warranted. Red indicates that the element is not functioning adequately and/or is not expected to effectively adapt to the shock to the system. See Figure 4 for an example scorecard. The radar plots in the last row show the scores of the individual indicators that roll-up to the lights displayed in the ‘what if’ scenarios in the rows above.

The scorecard is an important outcome in that it is a meaningful way to display aggregate results to the health system stakeholders in order to understand the areas in need of action. Therefore, scorecards must take into account that there may be various effective ways that a given health system may be able to absorb and/or adapt to a particular shock. Ultimately, the outcome of the resilience test determines if the system has enough green lights on (a) enough indicators, or (b) enough critical indictors, or (c) the right combination of indicators, to effectively respond to the shock. A health system that achieves green lights for all elements under all scenarios may want to assess alternate scenarios to ensure resiliency under different types of shocks. Green lights across all elements and scenarios might also suggest that this system is ready to further the integration of existing capacities and move towards becoming an integrated resilient health system.

The resilience test implementation process depends heavily on the collection of qualitative data via discussion groups with key stakeholders. This information, combined with available quantitative data, must be quantified according to the key indicators and used for score card generation. Once data collection in the resilience test implementation process has concluded in Phases 1 and 2 of the resilience test implementation process, the facilitator, in collaboration with an external support team and possibly with regional/national health authorities, will rate each indicator on a 4-point Likert scale, both for functioning under normal conditions and in each adverse scenario discussed. In other words, for each indicator, a forced choice as to adequacy vs. inadequacy must be made. This occurs in the summarization Phase 3.

In order to roll-up the multiple indicators of functioning into an overall stoplight value of a given MHSCS element, the indicators must be weighted. Ideally, weighting of critical indicators would be evidence based, using scientific literature to describe the relationship between similar shocks experienced in the past with their inputs/outputs/outcomes impacts and responses within a given health system. However, the health care system and its ecosystem is a complex system, and shocks can be expected to cause context-dependent impacts and context-specific responses. Thus, there are a number of significant challenges with an evidence-based approach to scoring, including the different characteristics of a health care system influencing the impact of the shock and the system’s response to the shock, the existence of multiple shocks in any given prior actual adverse scenario whose impacts cannot be teased apart, the role of timing and order of multiple shocks on impacts and health system responses, and the multiplicative (not solely additive) impacts and responses to these multiple shocks.

To address these challenges, the resilience test implementation process allows the stakeholders involved to determine how the key indicators need to be weighted to appropriately assess the MHSCS element. This occurs in the Phase 1 discussion groups of different stakeholders. The determination of appropriate weights for the indicators may involve the realist approach of CMO configurations [24,25] previously discussed, or might leverage other techniques, such as multi-criteria decision making, from the field of operations research. This process customizes the results of the resilience test, and when effectively carried out, can be expected to lead to findings that are more meaningful and actionable for the health system.

## 4. Discussion

We have developed a resilience test concept and methodology, and described key components of a toolkit and a 5-phased approach to implementation of resilience testing that can be adapted to individual health systems. A resilience test of a health system becomes valuable to decision makers in that system only when it can offer actionable, system-specific results that can transform the system and make it more resilient to future shocks or structural changes in the future. However, this can be challenging because health systems are diverse. The development of a resilience test applicable across different health systems needs to balance the standardization of the methodology with its adaptability to be tailored to individual health systems. For this reason, our proposed resilience test incorporates a qualitative approach to data collection, via table-top exercises and collaborative discussions of ‘what if’ scenarios that can be supplemented with quantitative data on relevant indicators. Given the lack of existing literature on the practical application of resilience testing of health systems, each toolkit component could be further developed. We dedicated the discussion to providing a roadmap for this future research.

Regarding component 1, which relates to “what if” scenarios, various types of shocks and structural changes are possible. A heuristic that classifies possible shocks to a health system based on four dimensions—time, expansion, origin, and impact—is provided as Supplementary Table S1. Other scenarios might be developed for shocks such as an earthquake, or water poisoning by an unknown biological agent that affects multiple organs and leads to death over time (slow burn of two months) or permanent mental health problems. Additional scenarios might address structural changes, such as a sudden budget cut in health care as a result of financial crisis because of the economic impact of coping with COVID-19, steadily increasing privatization of a health system that impacts accessibility, or a decrease in quality due to corruption. Future work could define and develop adverse “what if” scenarios following and expanding upon the example of the super-bug (see Table 2) to apply and further test the applicability of our methods.

Regarding component 2, which related to the menu of key indicators (see Table 4), various different indicators are possible. However, the table is not a comprehensive list. Future work could refine and/or expand the indicators of health system functioning within the context of a resilience test. Targeted scoping reviews could be used to leverage the large number of existing indicators in the literature [14,15,17,18]. Potential indicators could be tailored and mapped to each of the ‘what if’ scenarios, depending on their hypothesized effect on each MHSCS element. Supplementary Table S1 may be used to link the dimensions of a shock to the particular elements and/or mechanisms of action affected within a health system. CMO configurations may be helpful to determine additional, context-specific relevant indicators for the different ‘what if’ scenarios.

Regarding component 3, future work could elaborate the discussion guides to be used by the facilitator that would be aligned with the full set of ‘what if’ scenarios and complete menu of indicators. Future considerations might include examination of the possible existence of a one-to-one relationship between indicators of functioning from “normal” to “shock”, as well as questions to help the stakeholders evaluate the comprehensiveness of the menu of indicators for each relevant MHSCS element. Specific questions to address the value of each element within the health care system, to be used for weighed scores, are warranted. Similarly, regarding component 4, the process of customization of results into a scorecard requires testing and validation, for instance through pilot studies of the resilience test implementation process with extensive and continuous monitoring, along with evaluation and documentation of strengths and weaknesses. The creation of a detailed case example or case study to include the resilience testing methodology would be valuable to enhance understanding of how the methods described in this paper might apply to diverse health and social care systems.

There are a number of pragmatic open-ended implementation issues to be resolved with future research. Consideration can be given to the appropriate time durations for each phase of the resilience test and define adequate follow-up periods, as well as how often the test should be repeated. The level at which to conduct the resilience test (national vs. regional, depending on decision-making authority) and the role of federal bodies (such as the European Commission) in carrying out the test deserves further examination. Communication of results should also be explored with respect to the information provided to the public and the methods used. A better understanding of the widespread applicability of this resilience testing methodology to both high and low income countries’ health and social care systems is required.

## 5. Conclusions

In summary, to enable health system strengthening and transformation, it is critical for countries to test their health system’s resilience and have a test methodology that is balanced in terms of standardization and system-specific characteristics/needs. This paper has refined the definition of resilience and mapped it to a multidimensional framework for health and social care. We outlined the foundation for a 5-phase resilience test characterized by a collaborative, multi-stakeholder design. Participatory processes involve diverse individuals from the health ecosystem in the assessment and transformation stages. Four resilience testing toolkit components, along with their customization processes, have been presented. These provide a certain level of standardization of the testing process, while permitting health authorities the flexibility of adapting the tools to their context. Additional research is needed to fully elaborate these components and pilot the customization processes. Furthermore, research is needed to test and validate the full 5-phase resilience test implementation process, which represents a significant advancement in the operationalization of health system resilience.

The type of participatory processes involved in the proposed resilience testing methodology suggests there is value in engaging in collaborative processes across borders. Learning communities created at the international level could function as scientific communities to bring together, synthesize and share evidence on experiences with resilience testing to support harmonization in international approaches balanced with system specifics.

## Figures and Tables

**Table 1:** Four components of the resilience test toolkit.

Components of a Resilience Test Toolkit
1. Adverse “what if” scenarios
2. Menu of key indicators
3. Associated discussion guides
4. Assessment scorecard template

**Table 2:** Example of basic elements of a resilience test adverse scenario: a “super-bug” outbreak.

“Super-Bug” Outbreak ‘What If’ Scenario to Be Customized
“On any given day, about one in 31 hospital patients has at least one healthcare associated infection”, stated by CDC. The health system of country ‘x’ is characterized by [fill in specifics of the country]. The hospital sector has ‘y’ hospitals, which employ ‘L’ workers overall. Every year ‘n’ patients are admitted into hospitals for various treatments, totaling ‘w’ days of stay. In a recent census of health care-associated infections in hospitals, it was found that 30% [or any other high number] of patients were affected by a particular species of multi-resistant bacteria. The reported mortality rate is 15%. The mode of transmission of infection of this infection is not yet fully understood, though contact with contaminated surfaces seems to be dominant. Closure of affected areas to control the outbreak through extensive cleaning is deemed necessary by experts. This means a shutdown of an estimated 35% of hospital capacity for a period of 10–14 days. The cleaning process entails considerable additional costs, with a 50% chance of a second cleaning procedure being required depending on results following the first cleaning procedure. Closure of hospital facilities will prevent admission and outpatient visits by new patients to affected facilities. Health authorities have to decide how to best control the “superbug”. The main question is how to achieve such control and resume normal activity levels of health care providers with the lowest cost to the population.

**Table 3:** Concern by specific stakeholder type as related to the super-bug ‘what if’ scenario to be used for further customization.

**Type of Stakeholder**	**Issues to Consider to Customize the Scenario for Certain Stakeholders**
Hospital managers Senior clinicians	- Is it possible, given funding and capacity constraints, to interrupt activity for cleaning? - What are the consequences for different key stakeholder groups in the various scenarios? - Is there flexibility in finding alternative treatment settings, including use of ambulatory settings or primary care? - Is it necessary to implement new training for health workers or new processes, or do existing processes, such as cleaning, just need to be enforced? - What issues need to be considered regarding presentation of the situation to the public need?
Managers Clinicians Political decision makers Patient groups	- Might the closure of hospitals affect some groups more than others? - Which patient groups might be most affected? - What mitigating measures are needed?
Political decision makers Hospital managers Clinicians	- Should each hospital develop its own communication plan or should decisions be centralized in some way? - Who leads or coordinates efforts in this respect? - What information should be released to the public?

**Table 4:** Potential indicators for each MHSCS conceptual framework input/output elements.

MHSCS Conceptual Framework Inputs/Outputs Elements	Functions (Capacities)		
	Example Potential Indicators of Essential Functions— Use of Existing and Consistent Development of New Knowledge and Resources	Example Potential Indicators of Critical Functions Under Shock— Effective, Timely Use of Available Knowledge and Resources; Rapid Development of New Knowledge and Resources	Example Quantitative Measures
Health workforce	Trains qualified professionals Integrates different specialties and disciplines Addresses mental health of professionals	Re-assigns health professionals Engages in task shifting Expands responsibilities of health professionals	# different types of professionals per population # patients per medical professional # integration schemes covering different types of patients and health professionals
Community Carers	Trains qualified professionals Retains qualified professionals	Coordinates community carers Communicates with community carers	# community carers per population
Medicines	Availability of needed medicines Accesses needed medicines	Has flexibility in purchasing Scales up to population level	# medications stockpiled
Infrastructure	Has spare capacity of physical resources Has ability to adapt existing infrastructure Has telehealth infrastructure	Re-deploys physical resources Adapts physical resources	# hospital beds/population # ICU beds/population
Information systems	Utilizes an integrated inter-professional EMR Tracks population health via standardized data, i.e., EMRs, surveys Designs alert systems Identifies quality improvement needs	Leverages existing data for routine surveillance Identifies at-risk populations quickly	Real-time data lag estimate # data fields populated with useful aggregate data to inform public health
Governance	Engages in participatory leadership Coordinates decision making across hierarchies Incorporates effective models of governance Informs public in a transparent way Encourages accountability Fosters environment for collaboration and learning Real-time response and decision making Responsive to feedback	Adapts leadership and governance structure in an agile manner Allocates clearly decision-making power under stress Potentiates public health messaging Takes advantage of strengths of collaborators Timely response and decision making	n.a.
Financing	Balances funding mechanisms Has a revenue structure Has a set of rules for financing	Mobilizes financial resources	% increase in funds
Health services	Potentiates primary care services Provides sufficient coverage of health needs Provides sufficient mental health care coverage Integrates mental health care into other services	Supports primary care services Maintains access in line with health needs Ensures access to care for vulnerable groups Maintains access to mental health care	Waiting times for services Satisfaction ratings % of population without coverage
Health promotion	Engages in prevention activities Encourages inter-sectoral collaboration	Maintains health promotion activities Strengthens inter-sectoral collaborations	# collaborating organizations

# means “number of”.

## Supplementary Materials

The following are available online at https://www.mdpi.com/article/10.3390/ijerph18094742/s1, Table S1: A Heuristic: Four Dimensions of Classification of Shocks to a Health System.
