# Supplementary material for: Resilience Testing of Health Systems: How Can It Be Done?
Source: Int J Environ Res Public Health. 2021 Apr 29;18(9):4742. doi: 10.3390/ijerph18094742 (PMC8124463; doi:10.3390/ijerph18094742)
Supplement: Supplementary file 1 [file ijerph-18-04742-s001.zip › ijerph-1209936-supplementary.pdf]

Supplementary Table S1: A Heuristic: Four Dimensions of Classification of Shocks to a Health System

Dimension 1	Time	Dimension 2	Expansion	Dimension 3	Origin	Dimension 4	Impact
Onset	Rapid<br>Slow	Human being	Systematically<br>Locally	Creature	Human<br>Plant<br>Animal<br>Microbe / Virus	People	Individual<br>Population<br>Patients<br>Staff
Occurrence	Complete<br>Gradual	Geographical	Local<br>Regional<br>National<br>Continental<br>Global	Particle	Non-synthetic<br>Synthetic	Non-human life	Animal/Species<br>Plant
Continuity	Acute<br>Chronic	Elemental	Air<br>Water<br>Terra<br>Ice Vapor	Nature	Air<br>Water<br>Terra<br>Ice Vapor	Infrastructure	Technical<br>Logistical<br>Medical<br>Non-Medical<br>Pharmaceutical
						Systems	Society<br>Political<br>Health
